# Interpolation by Hankel Translates of a Basis Function: Inversion Formulas and Polynomial Bounds

**DOI:** 10.1155/2014/242750

**Published:** 2014-01-16

**Authors:** Cristian Arteaga, Isabel Marrero

**Affiliations:** Departamento de Análisis Matemático, Universidad de La Laguna, 38271 La Laguna (Tenerife), Spain

## Abstract

For *μ* ≥ −1/2, the authors have developed elsewhere a scheme for interpolation by Hankel translates of a basis function Φ in certain spaces of continuous functions *Y*_*n*_ (*n* ∈ *ℕ*) depending on a weight *w*. The functions Φ and *w* are connected through the distributional identity *t* ^4*n*^(*h*_*μ*_′Φ)(*t*) = 1/*w*(*t*), where *h*_*μ*_′ denotes the generalized Hankel transform of order *μ*. In this paper, we use the projection operators associated with an appropriate direct sum decomposition of the Zemanian space *ℋ*_*μ*_ in order to derive explicit representations of the derivatives *S*_*μ*_^*m*^Φ and their Hankel transforms, the former ones being valid when *m* ∈ *ℤ*_+_ is restricted to a suitable interval for which *S*_*μ*_^*m*^Φ is continuous. Here, *S*_*μ*_^*m*^ denotes the *m*th iterate of the Bessel differential operator *S*_*μ*_ if *m* ∈ *ℕ*, while *S*_*μ*_^0^ is the identity operator. These formulas, which can be regarded as inverses of generalizations of the equation (*h*_*μ*_′Φ)(*t*) = 1/*t* ^4*n*^ *w*(*t*), will allow us to get some polynomial bounds for such derivatives. Corresponding results are obtained for the members of the interpolation space *Y*_*n*_.

## 1. Introduction

The method of radial basis function interpolation has seen substantial developments, both theoretical and computational, and in applications; compare [1–3] and references therein. A radially symmetric function in Euclidean space ℝ^*d*^ can be identified with a function on the positive real axis. The *d*-dimensional Fourier transform of a radial function is also radial and reduces to a 1-dimensional Hankel transform of order *d*/2 − 1 [4, Theorem 3.3]. The natural convolution structure in the positive real axis is not that of a group but is given by the family of the so-called Bessel-Kingman hypergroups, depending on a parameter *μ* ≥ −1/2. The Kingman convolution is defined upon a generalized translation operator, known as Delsarte translation, and for *μ* = −1/2 coincides with the standard one. Recently [5, 6], the authors have benefited from the hypergroup structure in order to provide a new approach to the problem of radial basis function interpolation, which extends the usual scheme. Such an approach yields a greater variety of manageable kernels, which could be useful in handling mathematical models built upon classes of radial basis functions depending on the order *μ* and whose performance is expected to improve by suitably adjusting *μ*, as it happens, for instance, with the family of Matérn kernels in [7, Supplement, page 6]. The examples and numerical experiments included in [5] seem to support this view.

Our scheme actually considers a variant of the Delsarte translation, the so-called Hankel translation, in order to accommodate the usual definition of the Hankel integral transformation, namely,

(1) $$\begin{array}{c} \left(h_{\mu }\varphi \right)\left(x\right)=\overset{\infty }{\underset{0}{\int }}\varphi \left(t\right){𝒥}_{\mu }\left(xt\right)dt\;\left(x\in I\right), \end{array}$$

where *I* = ]0, *∞*[, *𝒥*_*μ*_(*z*) = *z* ^1/2^ *J*_*μ*_(*z*)  (*z* ∈ *I*) and *J*_*μ*_ denotes the Bessel function of the first kind and order *μ* ∈ ℝ.

Aiming to define the Hankel transformation in spaces of distributions, Zemanian [8] introduced the space *ℋ*_*μ*_ of all those smooth, complex-valued functions *φ* = *φ*(*x*)  (*x* ∈ *I*) such that

(2) νμ,r(φ)=max0≤k≤r supx∈I|(1+x2)r(x−1D)kx−μ−1/2φ(x)|<∞(r∈ℤ+).

Here, and in the sequel, *D* = *D*_*x*_ = *d*/*dx* and (*x* ^−1^ *D*)^*k*^ is the operator *x* ^−1^ *D* iterated *k* times (*k* ∈ *ℕ*) or the identity operator (*k* = 0). When topologized by the family of norms {*ν*_*μ*,*r*_}_*r*∈*ℤ*_+__, *ℋ*_*μ*_ becomes a Fréchet space where *h*_*μ*_ is an automorphism provided that *μ* ≥ −1/2. Then the generalized Hankel transformation *h*_*μ*_′, defined by transposition on the dual *ℋ*_*μ*_′ of *ℋ*_*μ*_, is an automorphism of *ℋ*_*μ*_′ when this latter space is endowed with either its weak* or its strong topology.

The space *𝒪* consists of all those smooth, complex-valued functions *θ* on *I* such that *θψ* ∈ *ℋ*_*μ*_ whenever *ψ* ∈ *ℋ*_*μ*_ and the linear operator *ψ* ↦ *θψ* is a continuous mapping of *ℋ*_*μ*_ into itself. This *𝒪* is also the space of multipliers of *ℋ*_*μ*_′, the corresponding multiplication operators being defined by transposition [9].

Denote by *L*_*μ*,*l*_^1^ the class of all those Lebesgue measurable functions *u* = *u*(*t*)  (*t* ∈ *I*) such that

(3) $$\begin{array}{c} \overset{a}{\underset{0}{\int }}\left|u\left(t\right)\right|t^{\mu +1/2}dt<\infty \;\left(a>0\right). \end{array}$$

The following spaces were introduced in [5].

**Definition 1**

Let *w* = *w*(*t*) > 0  (*t* ∈ *I*) be a continuous function, let

(4) $$\begin{array}{c} S_{\mu }=S_{\mu ,t}=t^{-\mu -1/2}D_{t}t^{2\mu +1}D_{t}t^{-\mu -1/2} \end{array}$$

be the Bessel differential operator, and let

(5) $$\begin{array}{c} Y_{n}=\left\{f\in {ℋ}_{\mu }^{\prime }:h_{\mu }^{\prime }S_{\mu }^{n}f\in L_{\mu ,l}^{1}\cap L_{\mu ,w}^{2}\right\}\;\;\;\left(n\in {\mathbb{Z}}_{+}\right), \end{array}$$

where *S*_*μ*_^0^ is the identity operator, *S*_*μ*_^*n*^  (*n* ∈ *ℕ*) is the operator *S*_*μ*_ iterated *n* times, and *L*_*μ*,*w*_^2^ stands for the class of all measurable functions *u* = *u*(*t*)  (*t* ∈ *I*) satisfying

(6) $$\begin{array}{c} {||u||}_{\mu ,w}={\left(\overset{\infty }{\underset{0}{\int }}{\left|u\left(t\right)\right|}^{2}w\left(t\right)t^{\mu +1/2}dt\right)}^{1/2}<\infty . \end{array}$$

A seminorm (norm if *n* = 0) is defined on *Y*_*n*_ by setting

(7) |f|n=||hμ′Sμnf||μ,w=(∫0∞|(hμ′Sμnf)(t)|2w(t)tμ+1/2dt)1/2 (f∈Yn).

The function *w* will be called a weight function for *Y*_*n*_.

In [5], for *n* ∈ *ℕ* and suitable conditions on the weight *w* related to the values of *n*, the spaces *Y*_*n*_ were shown to consist of continuous functions on *I*. Also, interpolants to *f* ∈ *Y*_*n*_ of the form

(8) $$\begin{array}{c} \left(Uf\right)\left(x\right)=\overset{m}{\underset{i=1}{\sum }}\alpha_{i}\left(\tau_{a_{i}}\Phi \right)\left(x\right)+\overset{n-1}{\underset{j=0}{\sum }}\beta_{j}p_{\mu ,j}\left(x\right)\;\left(x\in I\right) \end{array}$$

were obtained, where {*a*_1_,…, *a*_*m*_} ⊂ *I* is the set of interpolation points; Φ ∈ *ℋ*_*μ*_′ is a complex function defined on *I* (the so-called basis function), connected with *w* through the distributional identity

(9) $$\begin{array}{c} t^{4n}\left(h_{\mu }^{\prime }\Phi \right)\left(t\right)=\frac{1}{w\left(t\right)}; \end{array}$$

*p*_*μ*,*j*_(*x*) = *x* ^2*j*+*μ*+1/2^  (*j* ∈ *ℤ*_+_, 0 ≤ *j* ≤ *n* − 1) are Müntz monomials; *τ*_*z*_  (*z* ∈ *I*) denotes the Hankel translation operator of order *μ*; and *α*_*i*_, *β*_*j*_  (*i*, *j* ∈ *ℤ*_+_, 1 ≤ *i* ≤ *m*, 0 ≤ *j* ≤ *n* − 1) are complex coefficients.

In [5] the regularity results for the basis distribution Φ [5, Theorem 4.4] and the members of *Y*_*n*_ [5, Theorems 3.2 and 3.6] were achieved with the aid of the Lagrange interpolation projector onto the space of Müntz polynomials

(10) πμ,n−1=span{pμ,j(t)=t2j+μ+1/2  (t∈I):j∈ℤ+,0≤j≤n−1}.

In this paper we use, instead, the projectors associated with a suitable direct sum decomposition *ℋ*_*μ*_ = *ℋ*_*μ*,*n*_ ⊕ Π_*μ*,*n*_(*ρ*) (which will be described in Section 2) to obtain conditions guaranteeing the regularity of Φ, the distributions in *Y*_*n*_, and their *S*_*μ*_^*m*^-derivatives. In spite of the conditions obtained being stronger than those in [5], this new approach has the advantage of providing an explicit representation of these functions, their *S*_*μ*_^*m*^-derivatives, and their Hankel transforms, along with some polynomial bounds. The formulas for *S*_*μ*_^*m*^Φ hold when *m* ∈ *ℤ*_+_ ranges over a suitable interval and may be considered as inverses of generalizations of the equation

(11) $$\begin{array}{c} \left(h_{\mu }^{\prime }\Phi \right)\left(t\right)=\frac{1}{t^{4n}w\left(t\right)}, \end{array}$$

valid on *ℋ*_*μ*,2*n*_′.

The paper is organized as follows. In Section 2 we introduce the direct sum decomposition *ℋ*_*μ*_ = *ℋ*_*μ*,*n*_ ⊕ Π_*μ*,*n*_(*ρ*) along with the projection operators *P*_*n*_ onto Π_*μ*,*n*_(*ρ*) and *Q*_*n*_ onto *ℋ*_*μ*,*n*_.  Section 3 is devoted to studying the properties of their adjoints *P*_*n*_′ and *Q*_*n*_′. Our main results are in Section 5, where a Hankel inversion formula is presented in a general setting and then specialized to basis distributions and members of the interpolation space *Y*_*n*_.  Section 4 contains some auxiliary results of a rather technical nature.

Throughout the rest of this paper *n* ∈ *ℕ* will be fixed. The positive real axis will be always denoted by *I*, while *μ* will stand for a real number not less than −1/2 and *C* will represent a suitable positive constant, depending only on the opportune subscripts (if any), whose value may vary from line to line. Furthermore, we will adhere to the notations *ℤ*_+_ = *ℕ* ∪ {0} for the set of nonnegative integers and *𝒥*_*μ*_(*z*) = *z* ^1/2^ *J*_*μ*_(*z*)  (*z* ∈ *I*) for the function giving the kernel of the Hankel transformation *h*_*μ*_. The symbol *𝒞* (resp., *𝒞* ^*n*−1^) will denote the space of all continuous (resp., of class *n* − 1) functions on *I* (note that *𝒞* = *𝒞* ^0^). For the operational rules of the Hankel transformation that eventually might be required, both in the classical and the generalized senses, the reader is mainly referred to [10].

## 2. The Operators *P*_*n*_ and *Q*_*n*_

In this section, we introduce the direct sum decomposition *ℋ*_*μ*_ = *ℋ*_*μ*,*n*_ ⊕ Π_*μ*,*n*_(*ρ*) along with the projectors *P*_*n*_ onto Π_*μ*,*n*_(*ρ*) and *Q*_*n*_ onto *ℋ*_*μ*,*n*_. The main properties of these projectors are established.

We begin by recalling some definitions and results from [5] which will be needed in the sequel.

**Proposition 2**

Assume *φ* ∈ *ℋ*_*μ*_. Then, for every *m* ∈ *ℤ*_+_ one has

(12) $$\begin{array}{c} x^{-\mu -1/2}\varphi \left(x\right)=\overset{m}{\underset{j=0}{\sum }}a_{2j}x^{2j}+R_{2m}\left(x\right)\;\left(x\in I\right), \end{array}$$

where

(13) a2j=12jj!limz→0+(z−1D)jz−μ−1/2φ(z)(j∈ℤ+,  0≤j≤m),

and the remainder term satisfies

(14) |R2m(x)|≤Cmx2m+1×max0≤k≤m supz∈I|z2(m−k)+1(z−1D)2m−k+1z−μ−1/2φ(z)|(x∈I)

for some *C*_*m*_ > 0.

**Definition 3**

Let

(15) ℋμ,n={φ∈ℋμ:limx→0+(x−1D)jx−μ−1/2φ(x)=0(j∈ℤ+,  0≤j≤n−1)}.

The space *ℋ*_*μ*,*n*_ is endowed with the topology inherited from that of *ℋ*_*μ*_.

In view of Proposition 2, loosely speaking, one can say that *ℋ*_*μ*,*n*_ consists of all those *φ* ∈ *ℋ*_*μ*_ such that *x* ^−*μ*−1/2^ *φ*(*x*) has a Maclaurin series expansion starting at *x* ^2*n*^.

**Definition 4**

For *j* ∈ *ℤ*_+_, the distribution Λ_*j*_ ∈ *ℋ*_*μ*_′ is defined by

(16) 〈Λj,φ〉=(−1)jcμ,jlimx→0+(x−1D)jx−μ−1/2φ(x)(φ∈ℋμ),

where *c*_*μ*,*j*_ = 2^*μ*+*j*^Γ(*μ* + *j* + 1).

**Theorem 5**

The following hold:

- Given *j* ∈ *ℤ*_+_, one has *h*_*μ*_′Λ_*j*_ = *p*_*μ*,*j*_, where *p*_*μ*,*j*_(*t*) = *t* ^2*j*+*μ*+1/2^  (*t* ∈ *I*).
- The kernel of the operator *S*_*μ*_^*n*^ in *ℋ*_*μ*_′ is *π*_*μ*,*n*−1_.

Next we introduce new spaces and mappings.

**Definition 6**

By *𝒱*_*μ*,*n*_ we denote the space of all complex-valued functions *ϕ* ∈ *𝒞* ^*n*−1^ such that the limit

(17) $$\begin{array}{c} \underset{x\to 0+}{\lim}{\left(x^{-1}D\right)}^{j}x^{-\mu -1/2}\phi \left(x\right) \end{array}$$

exists for all *j* ∈ *ℤ*_+_, 0 ≤ *j* ≤ *n* − 1.

Note that the functionals Λ_*j*_ (*j* ∈ *ℤ*_+_,  0 ≤ *j* ≤ *n* − 1) are well defined on *𝒱*_*μ*,*n*_.

**Definition 7**

The space *ℋ*_*μ*,*n*,∗_ consists of all those *ρ* ∈ *ℋ*_*μ*_ such that

(18) $$\begin{array}{c} \underset{x\to 0+}{\lim}x^{-\mu -1/2}\rho \left(x\right)=1,\\ \underset{x\to 0+}{\lim}{\left(x^{-1}D\right)}^{j}x^{-\mu -1/2}\rho \left(x\right)=0\;\left(j\in \mathbb{N},\;1\leq j\leq n-1\right). \end{array}$$

Given *ρ* ∈ *ℋ*_*μ*,*n*,∗_, we set

(19) $$\begin{array}{c} \Pi_{\mu ,n}\left(\rho \right)=\left\{x^{-\mu -1/2}\rho \left(x\right)p\left(x\right):p\in \pi_{\mu ,n-1}\right\}. \end{array}$$

**Definition 8**

Let *ρ* ∈ *ℋ*_*μ*,*n*,∗_ be fixed. The mappings *P*_*n*_ : *𝒱*_*μ*,*n*_ → *𝒱*_*μ*,*n*_ and *Q*_*n*_ = **I** − *P*_*n*_ : *𝒱*_*μ*,*n*_ → *𝒱*_*μ*,*n*_ (**I** the identity operator) are, respectively, defined by

(20) (Pnφ)(x)=ρ(x)∑j=0n−1〈Lj,φ〉x2j (φ∈𝒱μ,n,  x∈I),(Qnφ)(x) =φ(x)−ρ(x)∑j=0n−1〈Lj,φ〉x2j (φ∈𝒱μ,n,  x∈I),

where

(21) $$\begin{array}{c} L_{j}=\frac{{\left(-1\right)}^{j}}{2^{j}j!c_{\mu ,j}}\Lambda_{j}\;\left(j\in {\mathbb{Z}}_{+},\;\;0\leq j\leq n-1\right). \end{array}$$

The next theorem proves some useful properties of the operators *P*_*n*_ and *Q*_*n*_ and also clarifies the relationship between *ℋ*_*μ*_ and *ℋ*_*μ*,*n*_. In what follows we will adopt the usual notation *𝒩*(*P*) and *ℛ*(*P*) for the kernel and range of a linear operator *P*. Recall that a projector or projection *P* of a vector space *X* is a linear transformation *P* : *X* → *X* which is idempotent, meaning that *P* ^2^ = *P*.

**Theorem 9**

Let *P*_*n*_, *Q*_*n*_ be as in Definition 8.

(i) *P*_*n*_ *and Q*_*n*_ *are continuous linear mappings from ℋ*_*μ*_ *into ℋ*_*μ*_.
(ii) *P*_*n*_ *and Q*_*n*_ *are projections into ℋ*_*μ*_ *which satisfy*
  (22) $$\begin{array}{c} 𝒩\left(P_{n}\right)=ℛ\left(Q_{n}\right)={ℋ}_{\mu ,n},\;\;ℛ\left(P_{n}\right)=𝒩\left(Q_{n}\right)=\Pi_{\mu ,n}\left(\rho \right). \end{array}$$
(iii) *ℋ*_*μ*_ = *ℋ*_*μ*,*n*_ ⊕ Π_*μ*,*n*_(*ρ*).
(iv) *Finally,*
  (23) ℋμ=hμ(ℋμ,n)⊕hμ[Πμ,n(ρ)]=hμ(ℋμ,n)⊕{∑j=0n−1aj(−Sμ)j(hμρ)     (aj∈ℂ,  j∈ℤ+,  0≤j≤n−1)}.

**Proof**

To prove (i), let *φ* ∈ *ℋ*_*μ*_ and let *r*, *m* ∈ *ℤ*_+_, with 0 ≤ *m* ≤ *r*. Then

(24) (1+x2)r(x−1D)mx−μ−1/2(Pnφ)(x) =(1+x2)r(x−1D)m[x−μ−1/2ρ(x)∑j=0n−1〈Lj,φ〉x2j] =∑j=0n−1〈Lj,φ〉(1+x2)r(x−1D)mx−μ−1/2x2jρ(x)(x∈I).

Since *ρ* ∈ *ℋ*_*μ*_ and all even polynomials lie in *𝒪* [10, Lemma 5.3-1], we may write

(25) supx∈I|(1+x2)r(x−1D)mx−μ−1/2(Pnφ)(x)| ≤∑j=0n−1supz∈I|(1+z2)r(z−1D)mz−μ−1/2z2jρ(z)|  ×|〈Lj,φ〉| (φ∈ℋμ).

Now it suffices to take into account that *L*_*j*_ ∈ *ℋ*_*μ*_′  (*j* ∈ *ℤ*_+_, 0 ≤ *j* ≤ *n* − 1).

Next, let *φ* ∈ *ℋ*_*μ*_. A simple manipulation yields

(26) (Pn2φ)(x)=ρ(x)∑j=0n−1〈Lj,Pnφ〉x2j=ρ(x)∑j=0n−1 ∑k=0n−1〈Lj,z,z2kρ(z)〉〈Lk,φ〉x2j(x∈I).

Since *ρ* ∈ *ℋ*_*μ*,*n*,∗_, for *j*, *k* ∈ *ℤ*_+_, 0 ≤ *j*,  *k* ≤ *n* − 1, we have

(27) 〈Lj,z,z2kρ(z)〉=12jj!limz→0+(z−1D)jz−μ−1/2z2kρ(z)=12jj!∑i=0j(ji)limz→0+[(z−1D)iz2k] ×limz→0+[(z−1D)j−iz−μ−1/2ρ(z)]=12jj!limz→0+(z−1D)jz2k=2kk!2jj!·2k−j(k−j)!limz→0+z2(k−j)={1,k=j0,k≠j.

Plugging (27) into (26),

(28) $$\begin{array}{c} \left(P_{n}^{2}\varphi \right)\left(x\right)=\rho \left(x\right)\overset{n-1}{\underset{j=0}{\sum }}\langle L_{j},\varphi \rangle x^{2j}=\left(P_{n}\varphi \right)\left(x\right)\;\left(x\in I\right). \end{array}$$

Therefore, *P*_*n*_ is a projection, and hence so is *Q*_*n*_.

Let us show that *P*_*n*_ : *ℋ*_*μ*_ → Π_*μ*,*n*_(*ρ*) is onto. If *φ*(*x*) = *x* ^−*μ*−1/2^ *ρ*(*x*)*p*(*x*) ∈ Π_*μ*,*n*_(*ρ*), with *p* ∈ *π*_*μ*,*n*−1_, then *φ* ∈ *ℋ*_*μ*_ and, using (27), it is easily seen that *P*_*n*_ *φ* = *φ*.

In view of Definition 8, it is apparent that *φ* ∈ *ℋ*_*μ*,*n*_ implies *P*_*n*_ *φ* = 0, so that *ℋ*_*μ*,*n*_ ⊂ *𝒩*(*P*_*n*_). To prove the reverse inclusion, take *φ* ∈ *𝒩*(*P*_*n*_). Then

(29) $$\begin{array}{c} \rho \left(x\right)\overset{n-1}{\underset{j=0}{\sum }}\langle L_{j},\varphi \rangle x^{2j}=0\;\left(x\in I\right). \end{array}$$

As *ρ* ∈ *ℋ*_*μ*,*n*,∗_ we have lim_*x*→0+_ *x* ^−*μ*−1/2^ *ρ*(*x*) = 1, and hence there exists *δ* > 0 such that *x* ^−*μ*−1/2^ *ρ*(*x*) > 0  (0 < *x* < *δ*). Consequently,

(30) $$\begin{array}{c} \rho \left(x\right)\overset{n-1}{\underset{j=0}{\sum }}\langle L_{j},\varphi \rangle x^{2j}=0\;\left(0<x<\delta \right) \end{array}$$

forces

(31) $$\begin{array}{c} \langle L_{j},\varphi \rangle =0\;\left(j\in {\mathbb{Z}}_{+},\;\;0\leq j\leq n-1\right). \end{array}$$

From (21) we conclude that *φ* ∈ *ℋ*_*μ*,*n*_.

Since *Q*_*n*_ = **I** − *P*_*n*_, we also have

(32) $$\begin{array}{c} 𝒩\left(Q_{n}\right)=ℛ\left(P_{n}\right)=\Pi_{\mu ,n}\left(\rho \right),\;\;ℛ\left(Q_{n}\right)=𝒩\left(P_{n}\right)={ℋ}_{\mu ,n}. \end{array}$$

With the map *P*_*n*_ being a projection of *ℋ*_*μ*_, we may write

(33) $$\begin{array}{c} {ℋ}_{\mu }=𝒩\left(P_{n}\right)⊕ℛ\left(P_{n}\right)={ℋ}_{\mu ,n}⊕\Pi_{\mu ,n}\left(\rho \right). \end{array}$$

From the fact that *h*_*μ*_ is an automorphism of *ℋ*_*μ*_ we infer

(34) $$\begin{array}{c} {ℋ}_{\mu }=h_{\mu }\left({ℋ}_{\mu }\right)=h_{\mu }\left({ℋ}_{\mu ,n}\right)⊕h_{\mu }\left[\Pi_{\mu ,n}\left(\rho \right)\right]. \end{array}$$

Let *p* ∈ *π*_*μ*,*n*−1_, *p*(*x*) = ∑_*j*=0_^*n*−1^ *a*_*j*_ *x* ^2*j*+*μ*+1/2^  (*x* ∈ *I*,  *a*_*j*_ ∈ *ℂ*,  *j* ∈ *ℤ*_+_,  0 ≤ *j* ≤ *n* − 1), and consider *x* ^−*μ*−1/2^ *ρ*(*x*)*p*(*x*) ∈ Π_*μ*,*n*_(*ρ*). Then

(35) $$\begin{array}{c} x^{-\mu -1/2}\rho \left(x\right)p\left(x\right)=\overset{n-1}{\underset{j=0}{\sum }}a_{j}x^{2j}\rho \left(x\right), \end{array}$$

so that

(36) $$\begin{array}{c} h_{\mu }\left[x^{-\mu -1/2}\rho \left(x\right)p\left(x\right)\right]=\overset{n-1}{\underset{j=0}{\sum }}a_{j}{\left(-S_{\mu }\right)}^{j}\left(h_{\mu }\rho \right). \end{array}$$

We thus conclude

(37) hμ[Πμ,n(ρ)]={∑j=0n−1aj(−Sμ)j(hμρ)(aj∈ℂ,  j∈ℤ+,  0≤j≤n−1)}.

This completes the proof.

## 3. The Distribution Adjoints of *P*_*n*_ and *Q*_*n*_

This section is devoted to studying the definition and properties of the distribution adjoints of *P*_*n*_ and *Q*_*n*_. A new space of functions must be introduced first.

**Definition 10**

Set

(38) 𝒦μ,n={φ∈𝒞n−1:limx→0+(x−1D)jφ(x)=0(j∈ℤ+, 0≤j≤n−1)}.

**Definition 11**

The adjoints *P*_*n*_′, *Q*_*n*_′ : *ℋ*_*μ*_′ → *ℋ*_*μ*_′ of *P*_*n*_, *Q*_*n*_ : *ℋ*_*μ*_ → *ℋ*_*μ*_ are defined by transposition:

(39) $$\begin{array}{c} \langle P_{n}^{\prime }u,\varphi \rangle =\langle u,P_{n}\varphi \rangle \;\left(u\in {ℋ}_{\mu }^{\prime },\;\;\varphi \in {ℋ}_{\mu }\right),\\ \langle Q_{n}^{\prime }u,\varphi \rangle =\langle u,Q_{n}\varphi \rangle \;\left(u\in {ℋ}_{\mu }^{\prime },\;\;\varphi \in {ℋ}_{\mu }\right). \end{array}$$

**Theorem 12**

The operators *P*_*n*_′ and *Q*_*n*_′ have the following properties:

(i) *P*_*n*_′* and Q*_*n*_′* are projections and* *P*_*n*_′ + *Q*_*n*_′ = **I**′*, the identity on ℋ*_*μ*_′.
(ii) *If u* ∈ *ℋ*_*μ*_′*, then*
  (40) $$\begin{array}{c} P_{n}^{\prime }u=\overset{n-1}{\underset{j=0}{\sum }}b_{j}\left(u\right)L_{j}, \end{array}$$
 where *b*_*j*_(*u*) = 〈*u*_*x*_, *x* ^2*j*^ *ρ*(*x*)〉  (*j* ∈ *ℤ*_+_,  0 ≤ *j* ≤ *n* − 1).
(iii) *ℛ*(*P*_*n*_′) = *𝒩*(*Q*_*n*_′) = *h*_*μ*_′(*π*_*μ*,*n*−1_) = span{Λ_*j*_ : *j* ∈ *ℤ*_+_, 0 ≤ *j* ≤ *n* − 1}.
(iv) *𝒩*(*P*_*n*_′) = *ℛ*(*Q*_*n*_′) = {*u* ∈ *ℋ*_*μ*_′ : 〈*u*_*x*_, *x* ^2*j*^ *ρ*(*x*)〉 = 0 (*j* ∈ *ℤ*_+_,  0 ≤ *j* ≤ *n* − 1)} = Π_*μ*,*n*_^⊥^(*ρ*).
(v) *ℋ*_*μ*_′ = Π_*μ*,*n*_^⊥^(*ρ*) ⊕ span{Λ_*j*_ : *j* ∈ *ℤ*_+_,  0 ≤ *j* ≤ *n* − 1}.
(vi) *ψu* = *ψ*(*Q*_*n*_′*u*)* whenever u* ∈ *ℋ*_*μ*_′* and ψ* ∈ *𝒪*∩*𝒦*_*μ*,*n*_.

**Proof**

Part (i) is a direct consequence of *P*_*n*_ and *Q*_*n*_ being projections of *ℋ*_*μ*_, with *P*_*n*_ + *Q*_*n*_ = **I** on *ℋ*_*μ*_:

(41) 〈(Pn′+Qn′)u,φ〉=〈u,(Pn+Qn)φ〉=〈u,Iφ〉=〈I′u,φ〉 (φ∈ℋμ).

To establish (ii), let *u* ∈ *ℋ*_*μ*_′. Then

(42) 〈Pn′u,φ〉=〈u,Pnφ〉=〈ux,ρ(x)∑j=0n−1〈Lj,φ〉x2j〉=∑j=0n−1〈Lj,φ〉〈ux,x2jρ(x)〉=∑j=0n−1bj(u)〈Lj,φ〉=〈∑j=0n−1bj(u)Lj,φ〉 (φ∈ℋμ),

where *b*_*j*_(*u*) = 〈*u*_*x*_, *x* ^2*j*^ *ρ*(*x*)〉  (*j* ∈ *ℤ*_+_,  0 ≤ *j* ≤ *n* − 1).

Since, distributionally, (*h*_*μ*_′Λ_*j*_)(*x*) = *x* ^2*j*+*μ*+1/2^ for all *j* ∈ *ℤ*_+_,  0 ≤ *j* ≤ *n* − 1 (Theorem 5), from (ii) and (21) it follows that

(43) $$\begin{array}{c} ℛ\left(P_{n}^{\prime }\right)\subset h_{\mu }^{\prime }\left(\pi_{\mu ,n-1}\right). \end{array}$$

Next we prove

(44) $$\begin{array}{c} h_{\mu }^{\prime }\left(\pi_{\mu ,n-1}\right)\subset 𝒩\left(Q_{n}^{\prime }\right). \end{array}$$

Take *u* ∈ *h*_*μ*_′(*π*_*μ*,*n*−1_), so that

(45) $$\begin{array}{c} u=\overset{n-1}{\underset{j=0}{\sum }}a_{j}\Lambda_{j}\;\left(a_{j}\in \mathbb{C},\;\;j\in {\mathbb{Z}}_{+},\;\;0\leq j\leq n-1\right), \end{array}$$

and let *φ* ∈ *ℋ*_*μ*_. Then *Q*_*n*_ *φ* ∈ *ℋ*_*μ*,*n*_ (Theorem 9) implies

(46) $$\begin{array}{c} \langle Q_{n}^{\prime }u,\varphi \rangle =\langle u,Q_{n}\varphi \rangle =\overset{n-1}{\underset{j=0}{\sum }}a_{j}\langle \Lambda_{j},Q_{n}\varphi \rangle =0. \end{array}$$

As *ℛ*(*P*_*n*_′) = *𝒩*(*Q*_*n*_′), by virtue of (43) and (44) we may write

(47) $$\begin{array}{c} ℛ\left(P_{n}^{\prime }\right)=𝒩\left(Q_{n}^{\prime }\right)=h_{\mu }^{\prime }\left(\pi_{\mu ,n-1}\right). \end{array}$$

On the other hand, given *u* ∈ *ℋ*_*μ*_′ we have *P*_*n*_′*u* = 0 if, and only if, *h*_*μ*_′(*P*_*n*_′*u*) = 0, or, from (ii) and (21),

(48) ∑j=0n−1bj(u)hμ′Lj=∑j=0n−1(−1)jbj(u)2jj!cμ,jhμ′Λj=∑j=0n−1(−1)jbj(u)2jj!cμ,jx2j+μ+1/2=0.

This happens if, and only if, 〈*u*_*x*_, *x* ^2*j*^ *ρ*(*x*)〉 = *b*_*j*_(*u*) = 0  (*j* ∈ *ℤ*_+_, 0 ≤ *j* ≤ *n* − 1). Therefore,

(49) R(Qn′)=𝒩(Pn′)={u∈ℋμ′:〈ux,x2jρ(x)〉=0  (j∈ℤ+,  0≤j≤n−1)}.

From the identity *P*_*n*_′ = **I**′ − *Q*_*n*_′ we arrive at

(50) ℋμ′=𝒩(Pn′)⊕ℛ(Pn′)={u∈ℋμ′:〈ux,x2jρ(x)〉=0(j∈ℤ+,  0≤j≤n−1)}⊕hμ′(πμ,n−1).

To complete the proof, let *φ* ∈ *ℋ*_*μ*_ and *ψ* ∈ *𝒪*∩*𝒦*_*μ*,*n*_. The Leibniz rule ensures that *ψφ* ∈ *ℋ*_*μ*,*n*_ = *ℛ*(*Q*_*n*_), so that *ψφ* = *Q*_*n*_(*ψφ*) (Theorem 9). Thus, for any *u* ∈ *ℋ*_*μ*_′ we may write

(51) 〈ψu,φ〉=〈u,ψφ〉=〈u,Qn(ψφ)〉=〈Qn′u,ψφ〉=〈ψ(Qn′u),φ〉.

The arbitrariness of *φ* ∈ *ℋ*_*μ*_ leads us to conclude that *ψu* = *ψ*(*Q*_*n*_′*u*) as distributions over *ℋ*_*μ*_.

## 4. Auxiliary Results

Here we prove two auxiliary lemmas.

**Lemma 13**

For *ψ* ∈ *ℋ*_*μ*_, there holds

(52) $$\begin{array}{c} P_{n}\left(h_{\mu }\psi \right)\left(t\right)=\overset{\infty }{\underset{0}{\int }}P_{n,\xi }\left({𝒥}_{\mu }\left(x\xi \right)\right)\left(t\right)\psi \left(x\right)dx\;\left(t\in I\right),\\ Q_{n}\left(h_{\mu }\psi \right)\left(t\right)=\overset{\infty }{\underset{0}{\int }}Q_{n,\xi }\left({𝒥}_{\mu }\left(x\xi \right)\right)\left(t\right)\psi \left(x\right)dx\;\left(t\in I\right). \end{array}$$

**Proof**

Let *ψ* ∈ *ℋ*_*μ*_ and fix *x* ∈ *I*. According to Definition 8, we have

(53) $$\begin{array}{c} P_{n,\xi }\left({𝒥}_{\mu }\left(x\xi \right)\right)\left(t\right)=\rho \left(t\right)\overset{n-1}{\underset{j=0}{\sum }}\langle L_{j,\xi },{𝒥}_{\mu }\left(x\xi \right)\rangle t^{2j}\;\left(t\in I\right), \end{array}$$

where

(54) 〈Lj,ξ,𝒥μ(xξ)〉=(−1)j2jj!cμ,j〈Λj,ξ,𝒥μ(xξ)〉=12jj!limξ→0+(ξ−1Dξ)jξ−μ−1/2(xξ)1/2Jμ(xξ)=xμ+1/22jj!limξ→0+(ξ−1Dξ)j(xξ)−μJμ(xξ)=(−1)jx2j+μ+1/22jj!limξ→0+(xξ)−μ−jJμ+j(xξ)=(−1)jx2j+μ+1/22jj!cμ,j.

Hence,

(55) $$\begin{array}{c} P_{n,\xi }\left({𝒥}_{\mu }\left(x\xi \right)\right)\left(t\right)=\rho \left(t\right)\overset{n-1}{\underset{j=0}{\sum }}\frac{{\left(-1\right)}^{j}x^{2j+\mu +1/2}}{2^{j}j!c_{\mu ,j}}t^{2j}\;\left(t\in I\right). \end{array}$$

Now,

(56) Pn(hμψ)(t)=ρ(t)∑j=0n−1〈Lj,hμψ〉t2j=ρ(t)∑j=0n−1(−1)j2jj!cμ,j〈Λj,hμψ〉t2j=ρ(t)∑j=0n−1(−1)j2jj!cμ,j〈hμ′Λj,ψ〉t2j=ρ(t)∑j=0n−1(−1)jt2j2jj!cμ,j∫0∞ψ(x)x2j+μ+1/2dx=∫0∞[ρ(t)∑j=0n−1(−1)jx2j+μ+1/22jj!cμ,jt2j]ψ(x)dx=∫0∞Pn,ξ(𝒥μ(xξ))(t)ψ(x)dx (t∈I).

Consequently

(57) Qn(hμψ)(t)=(hμψ)(t)−Pn(hμψ)(t)=∫0∞𝒥μ(xt)ψ(x)dx −∫0∞Pn,ξ(𝒥μ(xξ))(t)ψ(x)dx=∫0∞[𝒥μ(xt)−Pn,ξ(𝒥μ(xξ))(t)]ψ(x)dx=∫0∞Qn,ξ(𝒥μ(xξ))(t)ψ(x)dx (t∈I),

as asserted.

**Remark 14**

Since *ρ* ∈ *ℋ*_*μ*_ and all even polynomials lie in *𝒪*, from (55) it follows that

(58) $$\begin{array}{c} P_{n,\xi }\left({𝒥}_{\mu }\left(x\xi \right)\right)\in {ℋ}_{\mu }\subset {ℋ}_{\mu }^{\prime } \end{array}$$

for each *x* ∈ *I*. On the other hand, it is apparent that *𝒥*_*μ*_(*x*·) ∈ *ℋ*_*μ*_′ for each *x* ∈ *I*. Therefore, *Q*_*n*,*ξ*_(*𝒥*_*μ*_(*xξ*)) ∈ *ℋ*_*μ*_′ for each *x* ∈ *I*.

We close this section with some useful estimates.

**Lemma 15**

Suppose that the function *ρ* used to define the projection operator *Q*_*n*_ (cf. Definition 8) also satisfies 0 ≤ *t* ^−*μ*−1/2^ *ρ*(*t*) ≤ 1  (*t* ∈ *I*) and supp*ρ* ⊂ [0,1]. Then:

(i) for all *x*, *t* ∈ *I*, one has
  (59) |(x−1Dx)mx−μ−1/2[Qn,ξ(𝒥μ(xξ))(t)]| ≤C(1+x2)nt2m+μ+1/2,|Sμ,xm[Qn,ξ(𝒥μ(xξ))(t)]| ≤Cxμ+12(1+x2)n+m∑i=0mt2(m+i)+μ+1/2,
(ii) for *x* ∈ *I* and 0 < *t* ≤ *a*, one has
  (60) |(x−1Dx)mx−μ−1/2[Qn,ξ(𝒥μ(xξ))(t)]| ≤C(1+x2)nt2n+μ+1/2,
  (61) |Sμ,xm[Qn,ξ(𝒥μ(xξ))(t)]|≤Cxμ+1/2(1+x2)n+mt2n+μ+1/2.

**Proof**

Fix *x*, *t* ∈ *I*. Equation (55) gives

(62) Qn,ξ(𝒥μ(xξ))(t)=𝒥μ(xt)−Pn,ξ(𝒥μ(xξ))(t)=𝒥μ(xt)−ρ(t)∑j=0n−1(−1)jx2j+μ+1/22jj!cμ,jt2j,

so that

(63) (x−1Dx)mx−μ−1/2[Qn,ξ(𝒥μ(xξ))(t)]={(x−1Dx)mx−μ−1/2𝒥μ(xt) −ρ(t)∑k=0n−m−1(−1)m+kx2k2kk!cμ,m+kt2(m+k),m<n(x−1Dx)mx−μ−1/2𝒥μ(xt),m≥n.

If *m* ≥ *n*,

(64) (x−1Dx)mx−μ−1/2[Qn,ξ(𝒥μ(xξ))(t)] =(−1)mt2m+μ+1/2(xt)−μ−mJμ+m(xt),

whence

(65) $$\begin{array}{c} \left|{\left(x^{-1}D_{x}\right)}^{m}x^{-\mu -1/2}\left[Q_{n,\xi }\left({𝒥}_{\mu }\left(x\xi \right)\right)\left(t\right)\right]\right|\leq c_{\mu ,m}^{-1}t^{2m+\mu +1/2}. \end{array}$$

If *m* < *n*,

(66) (x−1Dx)mx−μ−1/2[Qn,ξ(𝒥μ(xξ))(t)] =(−1)mt2m+μ+1/2(xt)−μ−mJμ+m(xt)  −(−1)mt2mρ(t)∑k=0n−m−1(−1)kx2k2kk!cμ,m+kt2k,

whence

(67) |(x−1Dx)mx−μ−1/2[Qn,ξ(𝒥μ(xξ))(t)]| ≤(cμ,m−1+∑k=0n−m−1x2k2kk!cμ,m+k)t2m+μ+1/2 ≤[cμ,m−1+∑k=0n−m−1(1+x2)k2kk!cμ,m+k]t2m+μ+1/2 ≤C(1+x2)n−m−1t2m+μ+1/2.

Here we have used our hypotheses on *ρ*. To summarize,

(68) |(x−1Dx)mx−μ−1/2[Qn,ξ(𝒥μ(xξ))(t)]| ≤{C(1+x2)n−m−1t2m+μ+1/2,m<nCt2m+μ+1/2,m≥n.

Thus we find

(69) |(x−1Dx)mx−μ−1/2[Qn,ξ(𝒥μ(xξ))(t)]| ≤C(1+x2)nt2m+μ+1/2.

Assume now 0 < *t* ≤ *a*. When *m* ≥ *n*, (65) yields

(70) |(x−1Dx)mx−μ−1/2[Qn,ξ(𝒥μ(xξ))(t)]| ≤cμ,m−1a2m+μ+1/2t2m+μ+1/2a2m+μ+1/2 ≤cμ,m−1a2(m−n)t2n+μ+1/2=Ct2n+μ+1/2.

If *m* < *n*, (66) can be written as

(71) (x−1Dx)mx−μ−1/2[Qn,ξ(𝒥μ(xξ))(t)] =([1−t−μ−1/2ρ(t)](xt)−μ−mJμ+m(xt)+t−μ−1/2ρ(t)   ×[(xt)−μ−mJμ+m(xt)−∑k=0n−m−1(−1)k(xt)2k2kk!cμ+m,k])   ×(−1)mt2m+μ+1/2 =([1−t−μ−1/2ρ(t)](xt)−μ−mJμ+m(xt)   +t−μ−1/2ρ(t)∑k=n−m∞(−1)k(xt)2k2kk!cμ+m,k)(−1)mt2m+μ+1/2.

Since *ρ* ∈ *ℋ*_*μ*,*n*,∗_, from Proposition 2 it follows that

(72) |(x−1Dx)mx−μ−1/2[Qn,ξ(𝒥μ(xξ))(t)]| ≤C([1−t−μ−1/2ρ(t)]+t−μ−1/2ρ(t)(xt)2(n−m))t2m+μ+1/2 =C(t−μ−1/2[1−t−μ−1/2ρ(t)]t2nt2m+μ+1/2    +t−μ−1/2ρ(t)x2(n−m))t2n+μ+1/2 ≤C[1−t−μ−1/2ρ(t)t2na2m+t−μ−1/2ρ(t)]  ×(1+x2)n−mt2n+μ+1/2 ≤C(1+x2)n−mt2n+μ+1/2.

To summarize,

(73) |(x−1Dx)mx−μ−1/2[Qn,ξ(𝒥μ(xξ))(t)]| ≤{C(1+x2)n−mt2n+μ+1/2,m<nCt2n+μ+1/2,m≥n.

In any case, we get

(74) |(x−1Dx)mx−μ−1/2[Qn,ξ(𝒥μ(xξ))(t)]| ≤C(1+x2)nt2n+μ+1/2.

To complete the proof we note that if *ϕ* ∈ *𝒞* ^2*m*^, then

(75) $$\begin{array}{c} x^{-\mu -1/2}\left(S_{\mu }^{m}\phi \right)\left(x\right)=\overset{m}{\underset{i=0}{\sum }}a_{m,i}x^{2i}{\left(x^{-1}D\right)}^{m+i}x^{-\mu -1/2}\phi \left(x\right) \end{array}$$

for suitable coefficients *a*_*m*,*i*_  (*i* ∈ *ℤ*_+_, 0 ≤ *i* ≤ *m*). Hence

(76) |x−μ−1/2Sμ,xm[Qn,ξ(𝒥μ(xξ))(t)]| ≤C∑i=0mx2i(1+x2)nt2(m+i)+μ+1/2 ≤C(1+x2)n+m∑i=0mt2(m+i)+μ+1/2

for any *t* ∈ *I*, while

(77) |x−μ−1/2Sμ,xm[Qn,ξ(𝒥μ(xξ))(t)]| ≤C∑i=0mx2i(1+x2)nt2n+μ+1/2 ≤C(1+x2)n+mt2n+μ+1/2

if 0 < *t* ≤ *a* for some *a* ∈ *I*.

## 5. Main Results

Throughout this section we will assume that the function *ρ* used in Definition 8 satisfies 0 ≤ *x* ^−*μ*−1/2^ *ρ*(*x*) ≤ 1  (*x* ∈ *I*) and supp*ρ* ⊂ [0,1], so that Lemma 15 applies.

First we prove a regularity result, along with a Hankel inversion formula and a polynomial estimate, in a general setting.

**Theorem 16**

Let *f* ∈ *ℋ*_*μ*_′ be such that the distribution *h*_*μ*_′*f* is regular on *ℋ*_*μ*,*n*_. Then, for all *m* ∈ *ℤ*_+_,

(78) 〈hμ′(Sμmf),ψ〉=∫0∞(hμ′f)(ξ)(−ξ2)m(Qnψ)(ξ)dξ+〈hμ′(Sμmp),ψ〉 (ψ∈ℋμ),

where

(79) $$\begin{array}{c} p\left(\xi \right)=\overset{n-1}{\underset{j=0}{\sum }}\frac{{\left(-1\right)}^{j}\langle \left(h_{\mu }^{\prime }f\right)\left(x\right),x^{2j}\rho \left(x\right)\rangle }{2^{j}j!c_{\mu ,j}}\xi^{2j+\mu +1/2}\;\left(\xi \in I\right). \end{array}$$

Further, if there exists *r* ∈ *ℤ*_+_ and a function *G* integrable on *I* for which

(80) |(hμ′f)(t)Sμ,xm[Qn,ξ(𝒥μ(xξ))(t)]| ≤C(1+x2)rG(t) (x,t∈I),

where *C* is independent of *t*, then *S*_*μ*_^*m*^ *f* ∈ *𝒞*, with

(81) (Sμmf)(x)=∫0∞(hμ′f)(t)Sμ,xm[Qn,ξ(𝒥μ(xξ))(t)]dt+(Sμmp)(x) (x∈I),

and the estimate

(82) $$\begin{array}{c} \left|\left(S_{\mu }^{m}f\right)\left(x\right)\right|\leq C{\left(1+x^{2}\right)}^{r}+\left|\left(S_{\mu }^{m}p\right)\left(x\right)\right|\;\left(x\in I\right) \end{array}$$

holds.

**Proof**

Fix *m* ∈ *ℤ*_+_ and let *ψ* ∈ *ℋ*_*μ*_. We have

(83) 〈hμ′(Sμmf),ψ〉=〈(−ξ2)m(hμ′f)(ξ),ψ(ξ)〉=〈(hμ′f)(ξ),(−ξ2)mψ(ξ)〉=〈(hμ′f)(ξ),(−ξ2)m(Qnψ)(ξ)〉+〈(hμ′f)(ξ),(−ξ2)m(Pnψ)(ξ)〉.

On the one hand, from our hypotheses on *f* and since *Q*_*n*_ *ψ* ∈ *ℋ*_*μ*,*n*_, we may write

(84) 〈(hμ′f)(ξ),(−ξ2)m(Qnψ)(ξ)〉 =∫0∞(hμ′f)(ξ)(−ξ2)m(Qnψ)(ξ)dξ.

On the other hand,

(85) hμ′[Pn′(hμ′f)](ξ) =∑j=0n−1(−1)j〈(hμ′f)(x),x2jρ(x)〉2jj!cμ,jξ2j+μ+1/2=p(ξ)(ξ∈I),

or

(86) $$\begin{array}{c} P_{n}^{\prime }\left(h_{\mu }^{\prime }f\right)=h_{\mu }^{\prime }p. \end{array}$$

Thus,

(87) 〈(hμ′f)(ξ),(−ξ2)m(Pnψ)(ξ)〉 =〈(hμ′f)(ξ),Pn[(−t2)mψ(t)](ξ)〉 =〈(−ξ2)mPn′(hμ′f)(ξ),ψ(ξ)〉 =〈(−ξ2)m(hμ′p)(ξ),ψ(ξ)〉=〈hμ′(Sμmp),ψ〉.

A combination of (83), (84), and (87) gives (78).

Note that (80) and the fact that (*h*_*μ*_′*f*)*S*_*μ*,*x*_^*m*^[*Q*_*n*,*ξ*_(*𝒥*_*μ*_(*xξ*))] ∈ *𝒞* as a function of *x* ∈ *I* ensure that

(88) $$\begin{array}{c} \overset{\infty }{\underset{0}{\int }}\left(h_{\mu }^{\prime }f\right)\left(t\right)S_{\mu ,x}^{m}\left[Q_{n,\xi }\left({𝒥}_{\mu }\left(x\xi \right)\right)\left(t\right)\right]dt \end{array}$$

also belongs to *𝒞* as a function of *x* ∈ *I* (cf. [11, Proposition 7.8.3]). Furthermore, (80) entails

(89) ∫0∞|(hμ′f)(t)Sμ,xm[Qn,ξ(𝒥μ(xξ))(t)]|dt ≤C(1+x2)r (x∈I).

Now we have

(90) 〈Sμmf,ψ〉=〈hμ′f,hμ(Sμmψ)〉=〈hμ′f,Qn[hμ(Sμmψ)]〉+〈hμ′f,Pn[hμ(Sμmψ)]〉.

Lemma 13 allows us to write

(91) 〈hμ′f,Qn[hμ(Sμmψ)]〉 =∫0∞(hμ′f)(t)Qn[hμ(Sμmψ)](t)dt =∫0∞(hμ′f)(t)dt∫0∞Qn,ξ(𝒥μ(xξ))(t)(Sμmψ)(x)dx.

In view of Remark 14,

(92) ∫0∞Qn,ξ(𝒥μ(xξ))(t)(Sμmψ)(x)dx =〈Qn,ξ(𝒥μ(xξ))(t),(Sμmψ)(x)〉 =〈Sμ,xm[Qn,ξ(𝒥μ(xξ))(t)],ψ(x)〉 =∫0∞Sμ,xm[Qn,ξ(𝒥μ(xξ))(t)]ψ(x)dx.

Hence

(93) 〈hμ′f,Qn[hμ(Sμmψ)]〉 =∫0∞(hμ′f)(t)dt∫0∞Sμ,xm[Qn,ξ(𝒥μ(xξ))(t)]ψ(x)dx =∫0∞ψ(x)dx∫0∞(hμ′f)(t)Sμ,xm[Qn,ξ(𝒥μ(xξ))(t)]dt =〈∫0∞(hμ′f)(t)Sμ,xm[Qn,ξ(𝒥μ(xξ))(t)]dt,ψ(x)〉,

the change in the order of integration being justified by (89). Lastly, from (86),

(94) 〈hμ′f,Pn[hμ(Sμmψ)]〉=〈hμ′[Pn′(hμ′f)],Sμmψ〉=〈p,Sμmψ〉=〈Sμmp,ψ〉.

Equations (90), (93), and (94) lead us to (81), while the estimate (82) follows immediately from (89) and (81). The proof is thus complete.

At this point, let us formalize the definition of a basis distribution.

**Definition 17**

We call Φ ∈ *ℋ*_*μ*_′ a basis distribution if *t* ^4*n*^(*h*_*μ*_′Φ)(*t*) = 1/*w*(*t*) for some weight *w* (cf.  Definition 1) such that 1/*w* ∈ *L*_*μ*,*l*_^1^ and there exists *γ* ∈ ℝ with 1/*w*(*t*) = *O*(*t* ^−*γ*^) as *t* → *∞*.

The existence of basis distributions is guaranteed by the next Lemma 18, which also proves that a basis distribution is unique modulo a polynomial in *π*_*μ*,2*n*−1_. Although Lemma 18 is substantially Lemma  3.3 in [5], its proof illustrates the ideas behind our main results below, and we include it for the sake of completeness.

**Lemma 18**

Assume *F* ∈ *L*_*μ*,*l*_^1^ and there exists *γ* ∈ ℝ such that *F*(*x*) = *O*(*x* ^−*γ*^) as *x* → *∞*. Let *r* ∈ *ℤ*_+_. On *ℋ*_*μ*,*r*_ we define the linear functional *F*_*r*_ by

(95) $$\begin{array}{c} \langle F_{r},\psi \rangle =\overset{\infty }{\underset{0}{\int }}\frac{F\left(t\right)}{{\left(-t^{2}\right)}^{r}}\psi \left(t\right)dt\;\left(\psi \in {ℋ}_{\mu ,r}\right). \end{array}$$

Then

(i) *F*_*r*_ ∈ *ℋ*_*μ*,*r*_′.
(ii) *Any extension F*_*r*_^*e*^ ∈ *ℋ*_*μ*_′* of F*_*r*_ *to ℋ*_*μ*_ *satisfies*
  (96) $$\begin{array}{c} \langle {\left(-t^{2}\right)}^{r}F_{r}^{e}\left(t\right),\varphi \left(t\right)\rangle =\langle F,\varphi \rangle \;\left(\varphi \in {ℋ}_{\mu }\right). \end{array}$$
(iii) *If F*_2*r*_^*e*^ ∈ *ℋ*_*μ*_′* is an extension of F*_2*r*_ ∈ *ℋ*_*μ*,2*r*_′* to ℋ*_*μ*_ *, then*
  (97) $$\begin{array}{c} \langle {\left(-t^{2}\right)}^{r}F_{2r}^{e}\left(t\right),\psi \left(t\right)\rangle =\langle F_{r},\psi \rangle \;\left(\psi \in {ℋ}_{\mu ,r}\right). \end{array}$$
(iv) *If F*_*r*_^1^ *and F*_*r*_^2^ *are two extensions of F*_*r*_ *to ℋ*_*μ*_ *, then h*_*μ*_′(*F*_*r*_^1^) − *h*_*μ*_′(*F*_*r*_^2^) ∈ *π*_*μ*,*r*−1_.

**Proof**

Note that (ii) gives *h*_*μ*_′*S*_*μ*_^*r*^(*h*_*μ*_′*F*_*r*_^*e*^)(*t*) = (−*t* ^2^)^*r*^ *F*_*r*_^*e*^(*t*) = *F*(*t*). Therefore, part (iv) is a consequence of Theorem 5.

Let *a*, *C* > 0 be such that *F*(*x*) ≤ *Cx* ^−*γ*^  (*x* > *a*). To prove (i), take *ψ* ∈ *ℋ*_*μ*,*r*_ and write

(98) $$\begin{array}{c} \langle F_{r},\psi \rangle =\overset{a}{\underset{0}{\int }}\frac{F\left(t\right)}{{\left(-t^{2}\right)}^{r}}\psi \left(t\right)dt+\overset{\infty }{\underset{a}{\int }}\frac{F\left(t\right)}{{\left(-t^{2}\right)}^{r}}\psi \left(t\right)dt. \end{array}$$

Using Proposition 2 and the hypothesis that *F* ∈ *L*_*μ*,*l*_^1^, for the first integral we obtain

(99) |∫0aF(t)(−t2)rψ(t)dt| ≤∫0a|t−μ−1/2ψ(t)|t2r|F(t)|tμ+1/2dt ≤C[supz∈I|(z−1D)rz−μ−1/2ψ(z)|    +max0≤k≤r supz∈I|z2(r−k)+1(z−1D)2r−k+1z−μ−1/2ψ(z)|].

As to the second integral, we get

(100) |∫a∞F(t)(−t2)rψ(t)dt| ≤C∫a∞|tk−μ−1/2ψ(t)|tk+2r+γ−μ−1/2dt ≤C supz∈I|zk−μ−1/2ψ(z)|∫a∞dttk+2r+γ−μ−1/2 =C supz∈I|zk−μ−1/2ψ(z)|,

provided *k* ∈ *ℤ*_+_ is chosen so that *k* > −2*r* − *γ* + *μ* + 3/2. A combination of (98), (99), and (100) along with the arbitrariness of *ψ* ∈ *ℋ*_*μ*,*r*_ completes the proof of (i).

Now we establish (ii). First of all, we note that specializing *r* = 0 in (i) we obtain *F* ∈ *ℋ*_*μ*_′. Next, let *F*_*r*_^*e*^ ∈ *ℋ*_*μ*_′ be an extension of *F*_*r*_ to *ℋ*_*μ*_, and let *φ* ∈ *ℋ*_*μ*_. Since (−*t* ^2^)^*r*^ *φ*(*t*) ∈ *ℋ*_*μ*,*r*_, we may write

(101) 〈(−t2)rFre(t),φ(t)〉 =〈Fre(t),(−t2)rφ(t)〉=∫0∞F(t)(−t2)r(−t2)rφ(t)dt =∫0  ∞F(t)φ(t)dt=〈F,φ〉.

The arbitrariness of *φ* ∈ *ℋ*_*μ*_ gives (ii).

Finally, we prove (iii). Define *F*_2*r*_ ∈ *ℋ*_*μ*,2*r*_′ by (95), and let *F*_2*r*_^*e*^ ∈ *ℋ*_*μ*_′ be an extension of *F*_2*r*_ to *ℋ*_*μ*_. Then

(102) 〈(−t2)rF2re(t),ψ(t)〉 =〈F2re(t),(−t2)rψ(t)〉=∫0∞F(t)t4r(−t2)rψ(t)dt =∫0∞F(t)(−t2)rψ(t)dt=〈Fr,ψ〉

whenever *ψ* ∈ *ℋ*_*μ*,*r*_. Thus we are done.

When applied to a basis distribution Φ, Theorem 16 yields our second main result.

**Theorem 19**

Pick a weight function *w* with the properties that 1/*w* ∈ *L*_*μ*,*l*_^1^ and 1/*w*(*t*) = *O*(*t* ^−*γ*^) as *t* → *∞* for some *γ* ∈ ℝ. Let Φ ∈ *ℋ*_*μ*_′ satisfy *t* ^4*n*^(*h*_*μ*_′Φ)(*t*) = 1/*w*(*t*), so that *h*_*μ*_′Φ ∈ *ℋ*_*μ*,2*n*_′ and

(103) $$\begin{array}{c} \langle h_{\mu }^{\prime }\Phi ,\varphi \rangle =\overset{\infty }{\underset{0}{\int }}\frac{\varphi \left(t\right)}{t^{4n}w\left(t\right)}dt\;\left(\varphi \in {ℋ}_{\mu ,2n}\right) \end{array}$$

(Lemma 18). Then, for all *m* ∈ *ℤ*_+_ one has

(104) 〈hμ′(SμmΦ),ψ〉=(−1)m∫0∞ξ2(m−2n)(Q2nψ)(ξ)w(ξ)dξ+〈hμ′(Sμmp),ψ〉 (ψ∈ℋμ),

where

(105) $$\begin{array}{c} p\left(\xi \right)=\overset{2n-1}{\underset{j=0}{\sum }}\frac{{\left(-1\right)}^{j}\langle \left(h_{\mu }^{\prime }\Phi \right)\left(x\right),x^{2j}\rho \left(x\right)\rangle }{2^{j}j!c_{\mu ,j}}\xi^{2j+\mu +1/2}\;\left(\xi \in I\right). \end{array}$$

Further, if

(106) 4m<4n+γ−μ−32,

then *S*_*μ*_^*m*^Φ ∈ *𝒞*,

(107) (SμmΦ)(x)=∫0∞Sμ,xm[Q2n,ξ(𝒥μ(xξ))(t)]t4nw(t)dt+(Sμmp)(x) (x∈I),

and the inequality

(108) $$\begin{array}{c} \left|\left(S_{\mu }^{m}\Phi \right)\left(x\right)\right|\leq C{\left(1+x^{2}\right)}^{r}+\left|\left(S_{\mu }^{m}p\right)\left(x\right)\right|\;\left(x\in I\right) \end{array}$$

holds for some *r* ∈ *ℤ*_+_.

**Proof**

In order to derive this result from Theorem 16 it suffices to establish, under (106), an estimate like (80), with Φ instead of *f*.

Let *C* > 0,  *a* > 1 be such that 1/*w*(*t*) ≤ *Ct* ^−*γ*^  (*t* > *a*), with *γ* ∈ ℝ. Use Lemma 15 to choose *r* ∈ *ℤ*_+_ satisfying

(109) |Sμ,xm[Q2n,ξ(𝒥μ(xξ))(t)]|≤C(1+x2)rt4n+μ+1/2(x∈I, 0<t≤a),

(110) |Sμ,xm[Q2n,ξ(𝒥μ(xξ))(t)]|≤C(1+x2)r∑i=0mt2(m+i)+μ+1/2(x∈I,  t>a).

Note that

(111) (hμ′Φ)(t)Sμ,xm[Q2n,ξ(𝒥μ(xξ))(t)] =t4n(hμ′Φ)(t)Sμ,xm[Q2n,ξ(𝒥μ(xξ))(t)]t4n =Sμ,xm[Q2n,ξ(𝒥μ(xξ))(t)]t4nw(t) (x,t∈I).

Now

(112) |Sμ,xm[Q2n,ξ(𝒥μ(xξ))(t)]t4nw(t)| ≤C(1+x2)rt4n+μ+1/2t4nw(t) =C(1+x2)rtμ+1/2w(t) (x∈I)

if 0 < *t* ≤ *a*, while

(113) |Sμ,xm[Q2n,ξ(𝒥μ(xξ))(t)]t4nw(t)| ≤C(1+x2)r∑i=0mt2(m+i)+μ+1/2t4nw(t) ≤C(1+x2)rt4m−4n−γ+μ+1/2 (x∈I)

if *t* > *a*. Set

(114) $$\begin{array}{c} G\left(t\right)=\{\begin{array}{cc} \frac{t^{\mu +1/2}}{w\left(t\right)}, & 0<t\leq a\\ t^{4m-4n-\gamma +\mu +1/2}, & t>a. \end{array} \end{array}$$

This function is integrable on *I* as long as 1/*w* ∈ *L*_*μ*,*l*_^1^ and (106) holds. The required estimate is established by combining (111), (112), and (113).

**Remark 20**

In [5, Theorem 4.4], under the same hypotheses on the weight *w* as in Theorem 19, we proved that any basis distribution Φ has the property that *S*_*μ*_^*m*^Φ ∈ *𝒞* whenever *m* ∈ *ℤ*_+_ satisfies 2*m* < 4*n* + *γ* − *μ* − 3/2, a condition in fact weaker than (106). However, interestingly enough, Theorem 19 provides us with explicit expressions and polynomial bounds for the Bessel derivatives *S*_*μ*_^*m*^Φ whenever *m* ∈ *ℤ*_+_ satisfies (106).

Next we apply Theorem 16 to the distributions in *Y*_*n*_ in order to obtain Theorem 21, our last main result.

**Theorem 21**

Assume 1/*w* ∈ *L*_*μ*,*l*_^1^ and there exists *γ* ∈ ℝ such that 1/*w*(*t*) = *O*(*t* ^−*γ*^) as *t* → *∞*. Let *f* ∈ *Y*_*n*_, so that (−*t* ^2^)^*n*^(*h*_*μ*_′*f*)(*t*) ∈ *L*_*μ*,*l*_^1^. Then *h*_*μ*_′*f* ∈ *ℋ*_*μ*,*n*_′, and for all *m* ∈ *ℤ*_+_ one has

(115) 〈hμ′(Sμmf),ψ〉=∫0∞(hμ′f)(ξ)(−ξ2)m(Qnψ)(ξ)dξ+〈hμ′(Sμmp),ψ〉 (ψ∈ℋμ),

where

(116) $$\begin{array}{c} p\left(\xi \right)=\overset{n-1}{\underset{j=0}{\sum }}\frac{{\left(-1\right)}^{j}\langle \left(h_{\mu }^{\prime }f\right)\left(x\right),x^{2j}\rho \left(x\right)\rangle }{2^{j}j!c_{\mu ,j}}\xi^{2j+\mu +1/2}\;\left(\xi \in I\right). \end{array}$$

Further, if

(117) $$\begin{array}{c} 8m<4n+\gamma -\mu -\frac{3}{2}, \end{array}$$

then *S*_*μ*_^*m*^ *f* ∈ *𝒞*, with

(118) (Sμmf)(x)=∫0∞(hμ′f)(t)Sμ,xm[Qn,ξ(𝒥μ(xξ))(t)]dt+(Sμmp)(x) (x∈I),

and the estimate

(119) $$\begin{array}{c} \left|\left(S_{\mu }^{m}f\right)\left(x\right)\right|\leq C{\left(1+x^{2}\right)}^{r}+\left|\left(S_{\mu }^{m}p\right)\left(x\right)\right|\;\left(x\in I\right) \end{array}$$

holds for some *r* ∈ *ℤ*_+_.

**Proof**

Since *h*_*μ*_′(*S*_*μ*_^*n*^ *f*) ∈ *L*_*μ*,*w*_^2^, we have

(120) $$\begin{array}{c} {\left|f\right|}_{n}={\left(\overset{\infty }{\underset{0}{\int }}{\left|{\left(-\xi^{2}\right)}^{n}\left(h_{\mu }^{\prime }f\right)\left(\xi \right)\right|}^{2}w\left(\xi \right)\xi^{\mu +1/2}d\xi \right)}^{1/2}<\infty . \end{array}$$

Choose *C* > 0, *a* > 1 such that 1/*w*(*t*) ≤ *Ct* ^−*γ*^  (*t* > *a*), with *γ* ∈ ℝ. For any *ψ* ∈ *ℋ*_*μ*,*n*_, the Cauchy-Schwarz inequality gives

(121) ∫0∞|(hμ′f)(ξ)ψ(ξ)|dξ ≤|f|n(∫0∞|ξ−μ−1/2ψ(ξ)|2ξ4nw(ξ)ξμ+1/2dξ)1/2 ≤|f|n[(∫0a|ξ−μ−1/2ψ(ξ)|2ξ4nw(ξ)ξμ+1/2dξ)1/2     +(∫a∞|ξ−μ−1/2ψ(ξ)|2ξ4nw(ξ)ξμ+1/2dξ)1/2].

Using Proposition 2, for the first bounding integral we obtain

(122) ∫0a|ξ−μ−1/2ψ(ξ)|2ξ4nw(ξ)ξμ+1/2dξ ≤C[supz∈I|(z−1D)nz−μ−1/2ψ(z)|   +max0≤k≤n supz∈I|z2(n−k)+1(z−1D)2n−k+1z−μ−1/2ψ(z)|]2.

As to the second one,

(123) ∫a∞|ξ−μ−1/2ψ(ξ)|2ξ4nw(ξ)ξμ+1/2dξ ≤C supz∈I|zk−μ−1/2ψ(z)|2∫a∞dξξ2k+4n+γ−μ−1/2 =C supz∈I|zk−μ−1/2ψ(z)|2,

provided *k* ∈ *ℤ*_+_ is chosen so that 2*k* > −4*n* − *γ* + *μ* + 3/2. This proves that *h*_*μ*_′*f* ∈ *ℋ*_*μ*,*n*_′. Now (115) follows from (78).

To complete the proof it suffices to establish, under (117), an estimate like (80) and apply Theorem 16.

With this purpose, fix *x* ∈ *I*. As above, factorize

(124) $$\begin{array}{c} \left(h_{\mu }^{\prime }f\right)\left(t\right)S_{\mu ,x}^{m}\left[Q_{n,t}\left({𝒥}_{\mu }\left(xt\right)\right)\left(t\right)\right]=F\left(t\right)R\left(t\right)\;\left(t\in I\right), \end{array}$$

where

(125) $$\begin{array}{c} F\left(t\right)={\left(-t^{2}\right)}^{n}\left(h_{\mu }^{\prime }f\right)\left(t\right)w^{1/2}\left(t\right)t^{(\mu +1/2)/2}\;\left(t\in I\right),\\ R\left(t\right)=\frac{S_{\mu ,x}^{m}\left[Q_{n,t}\left({𝒥}_{\mu }\left(xt\right)\right)\left(t\right)\right]}{{\left(-t^{2}\right)}^{n}w^{1/2}\left(t\right)}t^{-(\mu +1/2)/2}\;\left(t\in I\right). \end{array}$$

Because of (120), *F* is square-integrable on *I*. On the other hand, Lemma 15 and the argument in the proof of Theorem 19 show that

(126) |R(t)|=|Sμ,xm[Qn,t(𝒥μ(xt))(t)]t2nw1/2(t)|t−(μ+1/2)/2≤C(1+x2)rH(t) (t∈I)

for some *r* ∈ *ℤ*_+_, where

(127) $$\begin{array}{c} H^{2}\left(t\right)=\{\begin{array}{cc} \frac{t^{\mu +1/2}}{w\left(t\right)}, & 0<t\leq a\\ t^{8m-4n-\gamma +\mu +1/2}, & t>a \end{array} \end{array}$$

is integrable on *I*, as far as 1/*w* ∈ *L*_*μ*,*l*_^1^ and (117) holds. Letting *G*(*t*) = |*F*(*t*)|*H*(*t*)  (*t* ∈ *I*), from (124) we find that *G* is integrable and

(128) |(hμ′f)(t)Sμ,xm[Qn,t(𝒥μ(xt))(t)]| ≤C(1+x2)rG(t) (t∈I).

This ends the proof.

**Remark 22**

In [5, Theorem 3.2], under the same hypotheses on the weight *w* as in Theorem 21, we proved that every *f* ∈ *Y*_*n*_ has the property that *S*_*μ*_^*m*^ *f* ∈ *𝒞* whenever *m* ∈ *ℤ*_+_ satisfies 4*m* < 4*n* + *γ* − *μ* − 3/2. Although this condition is actually weaker than (117), Theorem 21 provides us with explicit expressions and polynomial bounds for those *S*_*μ*_-derivatives.
